# Characterization of the *Klebsiella pneumoniae* Secretome Using Size-Exclusion Chromatography and Raman Spectroscopy

**DOI:** 10.3390/ijms27093797

**Published:** 2026-04-24

**Authors:** Elizaveta Denisova, Anastasia Avdyusheva, Elizaveta Tyshchuk, Polina Grebenkina, Andrey Korenevsky, Ivan Chelibanov, Vladimir Chelibanov, Areg Totolian, Lyudmila Kraeva, Vitaly Nazarov, Dmitry Sokolov

**Affiliations:** 1 Saint-Petersburg Pasteur Institute, St. Petersburg 197101, Russia; liza.denisova9898@yandex.ru (E.D.); lizatyshchuk@yandex.ru (E.T.); grebenkinap@gmail.com (P.G.); a.korenevsky@yandex.ru (A.K.); ichelibanov@gmail.com (I.C.); totolian@pasteurorg.ru (A.T.); lykraeva@yandex.ru (L.K.); 2 Research Institute of Obstetrics and Gynecology Named After D.O. Ott, St. Petersburg 199034, Russia; avdyusheva04@mail.ru; 3 «OPTEC» Joint Stock Company, St. Petersburg 199178, Russia; chelibanov@gmail.com; 4 Military Medical Academy Named After S.M. Kirov, 6 Akademika Lebedeva Street, Building 6, St. Petersburg 194044, Russia; 5 Department of Faculty Surgery, North-Western State Medical University Named After I. I. Mechnikov, 41 Kirochnaya Street, St. Petersburg 191015, Russia; venazarov@yandex.ru

**Keywords:** ESKAPE pathogens, *Klebsiella pneumoniae*, bacterial secretome, Raman spectroscopy, chromatographic fractionation, size-exclusion chromatography, virulence metabolites

## Abstract

The secretome of ESKAPE pathogens, including *Klebsiella pneumoniae*, comprises a diverse array of bioactive molecules that govern virulence, antibiotic resistance, and the establishment of an immunosuppressive microenvironment. However, the high chemical complexity of the secretome impedes the identification of key metabolites mediating pathogenesis. In this study, we profiled the metabolite composition of cell-free *K. pneumoniae* supernatant using a combined approach of chromatographic fractionation and Raman spectroscopy. Chromatographic separation enabled the resolution of the complex secretome and revealed fractions with distinct biochemical signatures. A key finding was the identification of Fraction 3, characterized by a unique metabolic profile: it was enriched in nucleic acid fragments, peptides containing tyrosine and methionine, polysaccharides, and stress-response metabolites (e.g., citrate), while notably lacking markers of tryptophan and sterol-like lipids. These spectral signatures suggest a potential role for Fraction 3 metabolites in intercellular communication, biofilm formation, and protection against oxidative stress. The remaining fractions also exhibited distinct biochemical profiles, defined by unique profiles of lipids, nucleotides, and amino acids. Collectively, these data underscore the critical role of specific *K. pneumoniae* secreted metabolites to pathogen survival and host immune modulation. The combined approach effectively resolves functionally relevant secretome fractions, offering new avenues for identifying diagnostic and therapeutic targets for multidrug-resistant infections.

## 1. Introduction

Multidrug-resistant infections represent a major threat to global public health in the 21st century. ESKAPE pathogens (*Enterococcus faecium*, *Staphylococcus aureus*, *Klebsiella pneumoniae*, *Acinetobacter baumannii*, *Pseudomonas aeruginosa*, and *Enterobacter* spp.) dominate the landscape of nosocomial infections due to their ability to rapidly acquire resistance genes, form biofilms, and persist in hospital environments. This global burden is exemplified by *K. pneumoniae*, which is estimated to cause 50,000–100,000 deaths annually worldwide due to drug-resistant strains [1].

*K. pneumoniae* is an encapsulated, Gram-negative opportunistic pathogen responsible for both community- and healthcare-associated infections, including urinary tract infections, sepsis, bacteremia, soft tissue infections, and severe manifestations such as necrotizing pneumonia, pyogenic liver abscesses, and endogenous endophthalmitis [2,3]. Colonization of various host niches represents the initial step in infection development [3]. Although considered opportunistic, *K. pneumoniae* is part of the normal gut microbiota in approximately one-third of the population [4,5]. Of particular clinical concern is its intrinsic resistance to penicillin and frequent acquisition of multidrug resistance to β-lactam antibiotics, including carbapenems [4,5]. Strains are further classified into classical (cKp) and non-classical (ncKp) subtypes, which differ in virulence and resistance profiles [5].

The pathogenesis of *K. pneumoniae* infections is driven not only by genetic determinants of antibiotic resistance but also by a complex repertoire of secreted virulence factors [3,4,5,6,7,8,9]. Cell-free supernatants (secretomes) contain hundreds of bioactive molecules, including siderophores, capsular polysaccharides, toxins, outer membrane vesicles (OMVs), peptides, and metabolites, that modulate host immune responses, promote adhesion and invasion, and foster an immunosuppressive microenvironment [4,6,7,8,10]. For instance, *K. pneumoniae* produces diverse exopolysaccharides and enzymes involved in stress adaptation and antibiotic resistance [10]. Its secretome can suppress the oxidative burst in phagocytes, inhibit apoptosis of infected cells, and disrupt epithelial barrier integrity, thereby facilitating systemic dissemination [11,12].

Despite its clinical significance, the *K. pneumoniae* secretome, particularly its non-proteinaceous metabolites, remains poorly characterized [5]. Analyzing such a chemically complex mixture poses significant methodological challenges, as conventional analytical platforms often lack the selectivity and sensitivity required to detect low-abundance components within complex biological matrices [13].

Thus, comprehensive molecular characterization of the *K. pneumoniae* secretome constitutes a critical interdisciplinary endeavor. Elucidating how *K. pneumoniae* metabolites interact with the host immune system is essential for developing novel strategies to combat antibiotic-resistant infections, such as targeting key virulence factors or implementing immunomodulatory interventions.

To overcome the limitations of Raman spectroscopy in analyzing complex biological mixtures—primarily spectral interference from overlapping signals—a hybrid approach combining chromatographic fractionation to reduce sample complexity with subsequent Raman detection has been proposed [14,15,16,17,18,19].

In this study, we applied this integrated strategy to analyze the secretome of *K. pneumoniae*, a clinically relevant ESKAPE pathogen, with the aim of identifying functionally significant fractions potentially harboring key virulence or signaling molecules.

## 2. Results

**Figure A1:**
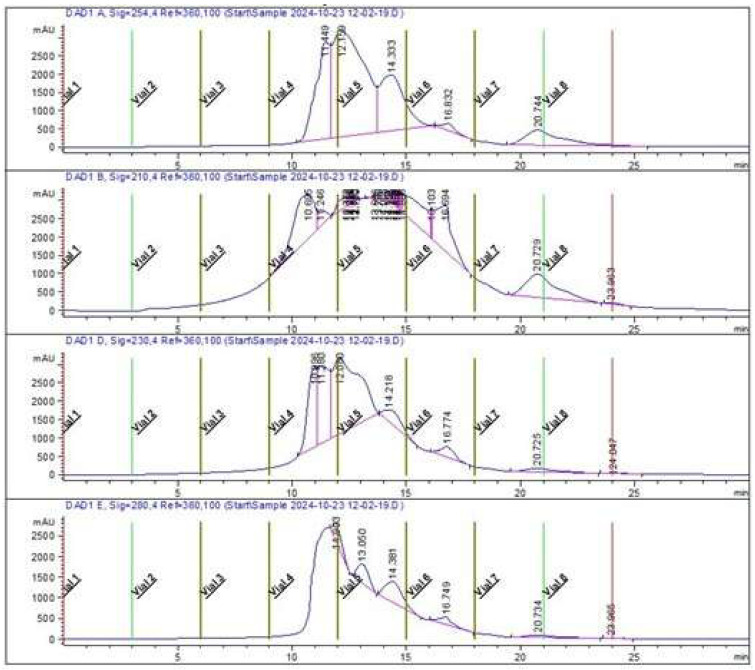
DAD detector chromatograms at wavelengths of 254.4 nm (**A**), 210.4 nm (**B**), 230.4 nm (**D**), and 280.4 nm (**E**), obtained during preparative separation of protein fractions from bacterial supernatants by gel filtration on a Bio SEC-3 column (Agilent). Peak retention times are indicated above the corresponding peaks. Samples: Vial 1—concentrated input supernatant; Vials 2–8—sequential fractions collected in time-line mode at 3-min intervals (Fractions 1–7, respectively). Analysis was performed under isocratic conditions at 4 °C, with a flow rate of 0.35 mL/min and distilled water as the mobile phase. Fractions collected after 24 min contained no detectable protein and were not further analyzed.

**Figure A2:**
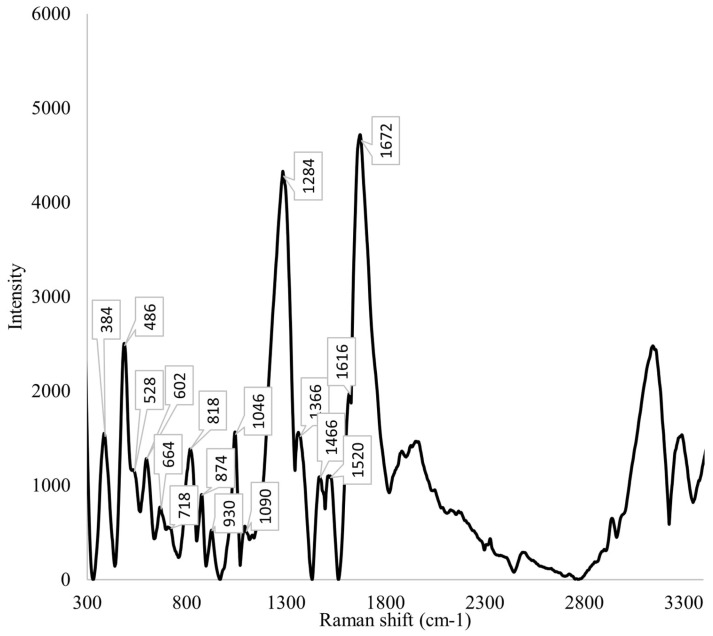
Raman spectrum of the whole cell-free supernatant of *Klebsiella pneumoniae* in the range of 300–3400 cm^−1^.

**Figure A3:**
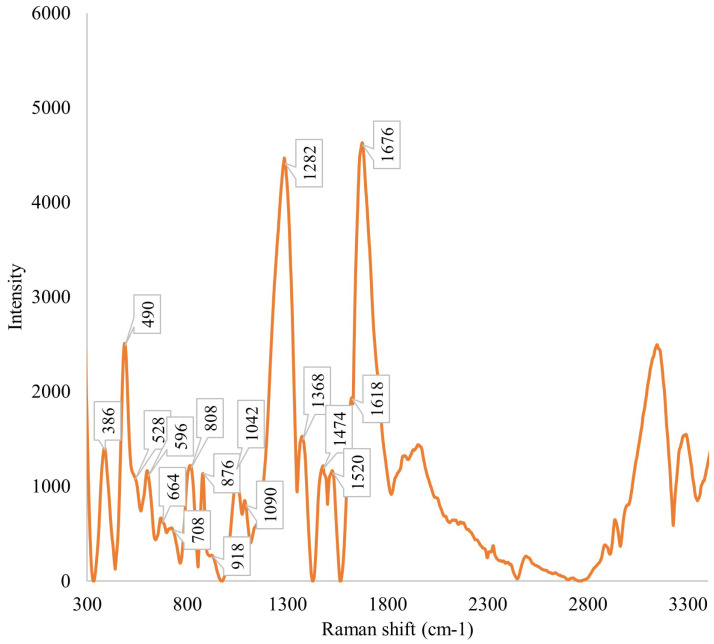
Raman spectrum of Fraction 1 of *Klebsiella pneumoniae* in the range of 300–3400 cm^−1^.

**Figure A4:**
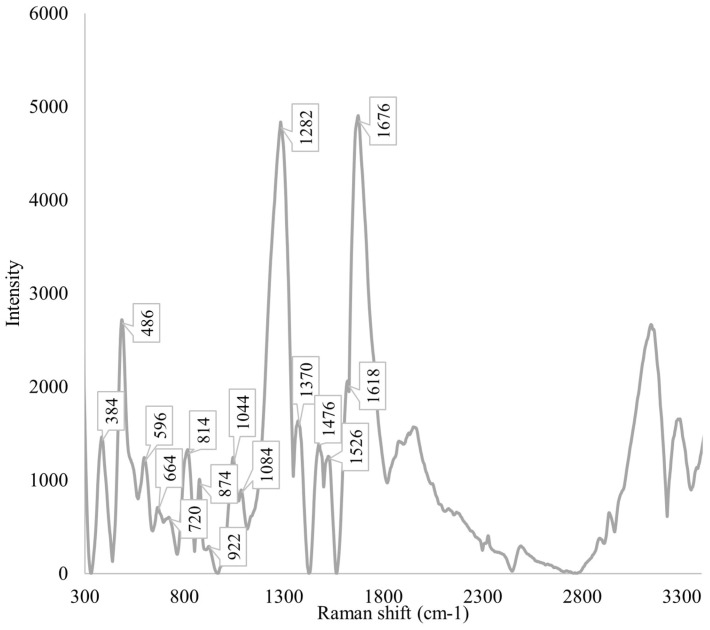
Raman spectrum of Fraction 2 of *Klebsiella pneumoniae* in the range of 300–3400 cm^−1^.

**Figure A5:**
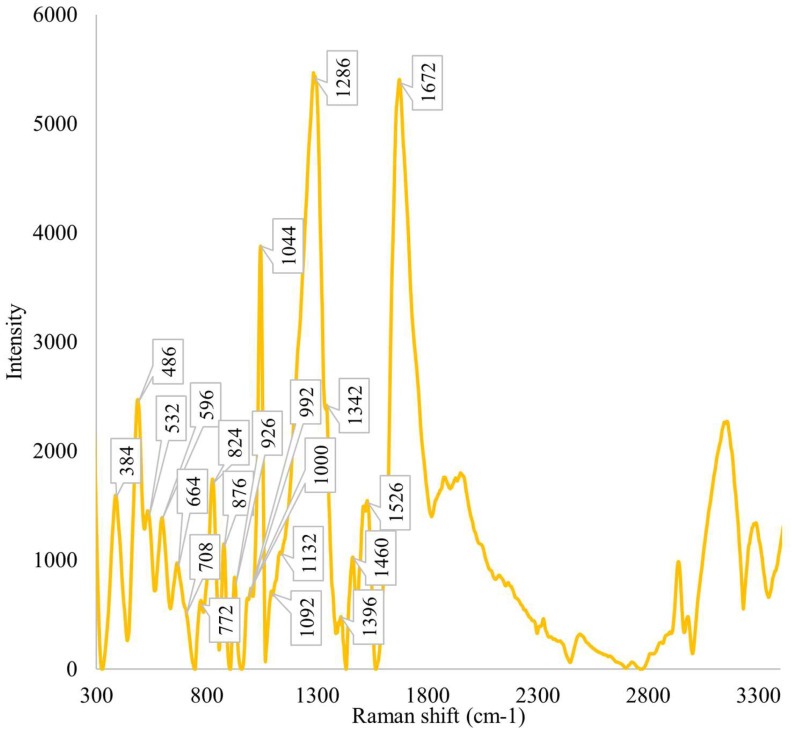
Raman spectrum of Fraction 3 of *Klebsiella pneumoniae* in the range of 300–3400 cm^−1^.

**Figure A6:**
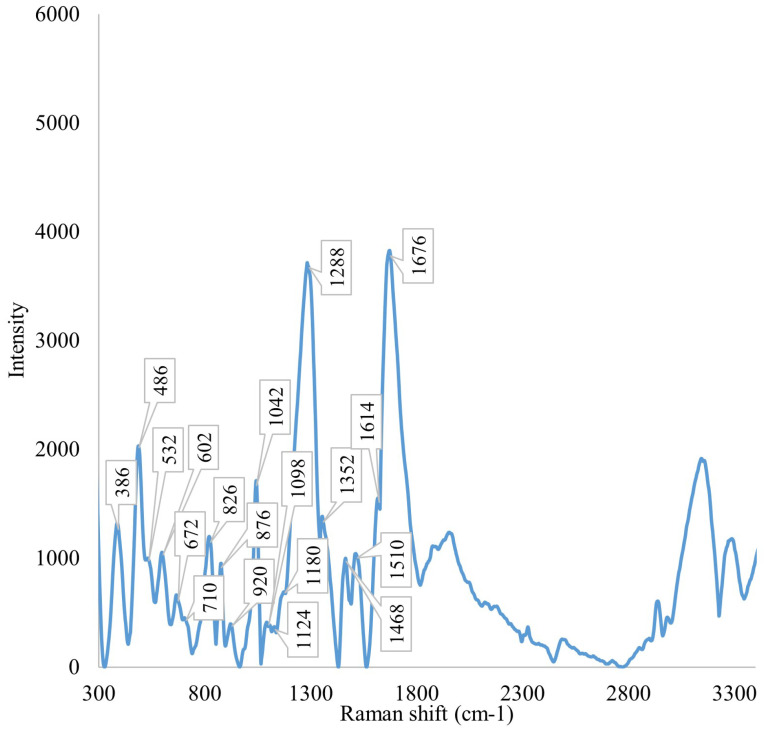
Raman spectrum of Fraction 4 of *Klebsiella pneumoniae* in the range of 300–3400 cm^−1^.

**Figure A7:**
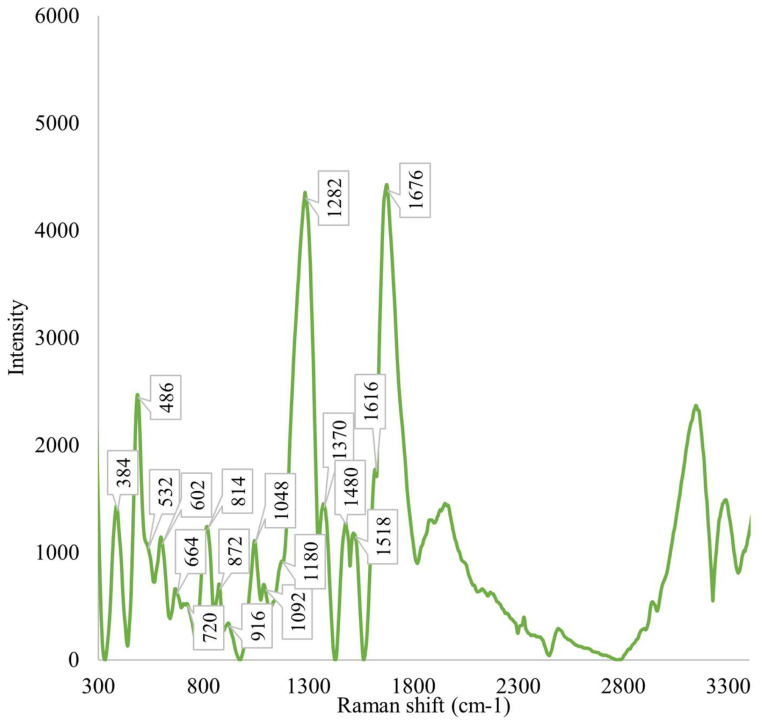
Raman spectrum of Fraction 5 of *Klebsiella pneumoniae* in the range of 300–3400 cm^−1^.

**Figure A8:**
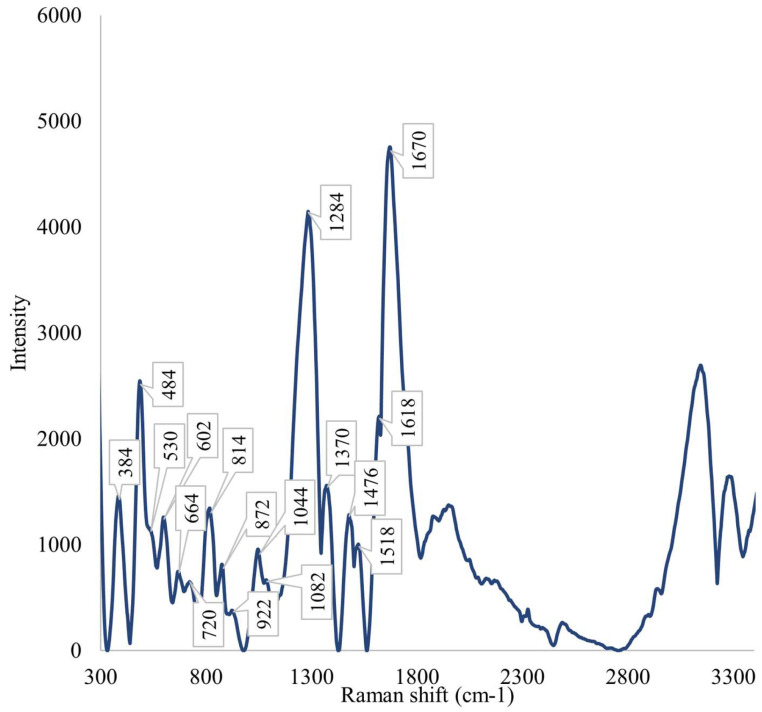
Raman spectrum of Fraction 6 of *Klebsiella pneumoniae* in the range of 300–3400 cm^−1^.

**Figure A9:**
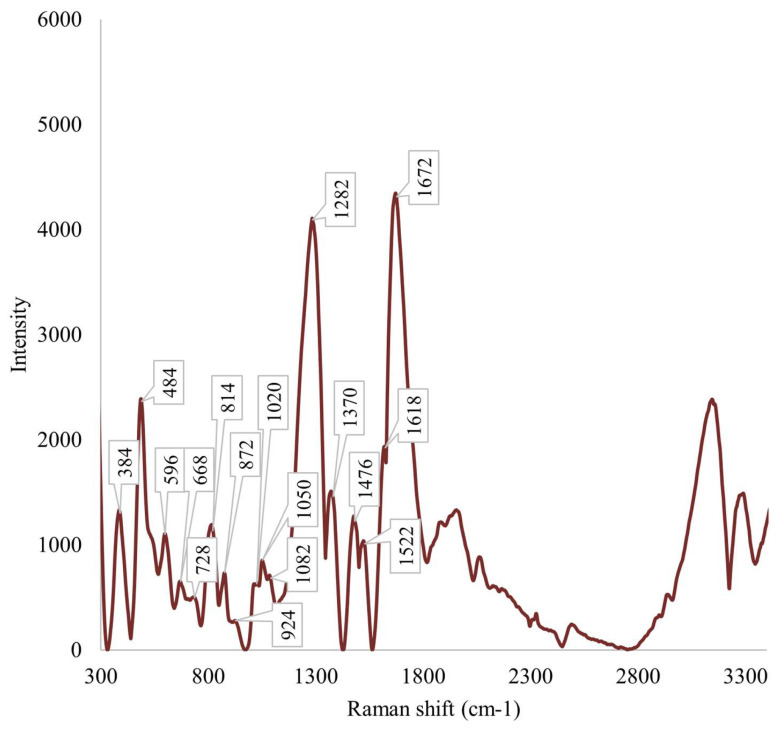
Raman spectrum of Fraction 7 of *Klebsiella pneumoniae* in the range of 300–3400 cm^−1^.

Following chromatographic fractionation (Figure A1) of the cell-free *K. pneumoniae* supernatant into seven fractions and acquisition of Raman spectra across the 300–3400 cm^−1^ range for all seven fractions and medium, the unfractionated supernatant (US), both common and unique spectral features were identified (Table 1; Figure A2, Figure A3, Figure A4, Figure A5, Figure A6, Figure A7, Figure A8 and Figure A9).

Intense Raman peaks at ~1670–1676 cm^−1^, ~1510–1524 cm^−1^, ~1282–1286 cm^−1^, ~1090–1098 cm^−1^, ~1082–1084 cm^−1^, ~1042–1050 cm^−1^, ~916–926 cm^−1^, ~814–826 cm^−1^, ~704–732 cm^−1^, ~664–672 cm^−1^, and ~384–386 cm^−1^ were consistently detected across all samples, including fractions 1–7 and the US (Table 1). These signals were present in all measurements and may be defined as core spectral markers of the *K. pneumoniae* metabolite profile.

Fraction 3 exhibited the most intense and complex spectral signature, characterized by a series of unique or significantly enhanced peaks compared to the other fractions and the US. Characteristic signals included the following (Table 1; Figure A5): ~1672 cm^−1^, ~1510–1526 cm^−1^, ~1446 cm^−1^, ~1382–1410 cm^−1^, ~1342 cm^−1^, ~1286 cm^−1^, ~1132 cm^−1^, ~1090–1098 cm^−1^, ~1082–1084 cm^−1^, ~1044 cm^−1^, ~992 cm^−1^, ~1000 cm^−1^, ~924 cm^−1^, ~876 cm^−1^, ~814 cm^−1^, ~772 cm^−1^, ~664 cm^−1^, ~596 cm^−1^, ~532 cm^−1^, ~486–490 cm^−1^, and ~384–386 cm^−1^.

The peak at ~1672 cm^−1^ was present in all fractions but reached maximum intensity in Fraction 3. Similarly, signals at ~1286 cm^−1^, ~1080 cm^−1^, and ~1044 cm^−1^ were observed in all samples, yet attained their highest intensities in Fraction 3. The peak at ~1044 cm^−1^ also showed a weak but discernible signal in Fraction 7. The band at ~1446 cm^−1^, observed in the spectrum of *K. pneumoniae* Fraction 3, exhibited markedly higher intensity compared to all other fractions, appearing only faintly in Fraction 4 and the US cell-free supernatant, and being absent or minimally expressed in the remaining fractions.

Notably, several peaks were exclusively absent in Fraction 3. The signal at ~1614–1618 cm^−1^ was detected in all fractions except Fraction 3. Additionally, a pronounced peak at ~880 cm^−1^ was observed in all *K. pneumoniae* fractions except Fractions 3, 5, 7, and the US. In these latter samples, this signal was either absent or substantially reduced in intensity; instead, a peak at ~876 cm^−1^ appeared, which was not detected in the other fractions. Likewise, peaks at ~614 cm^−1^, ~602 cm^−1^, ~596 cm^−1^, ~548 cm^−1^, and ~496 cm^−1^ were found in all fractions except Fractions 3 and 4.

Particular attention should be paid to the spectral region ~682–692 cm^−1^. A distinct peak at ~686 cm^−1^ was present in the spectrum of the US but absent in all chromatographic fractions. Conversely, a peak at ~688 cm^−1^ was observed in Fractions 6 and 7, but not in Fractions 3 and 4 (Figure A5 and Figure A6). Furthermore, a unique peak at ~930 cm^−1^ was detected only in the US and was completely absent in all fractionated samples. In contrast, the peak at ~924 cm^−1^ was observed exclusively in Fraction 3.

Fraction 2 was distinguished by a unique, intense peak at ~486 cm^−1^, absent in all other fractions and the US (Figure A4). Fractions 4 and 5 shared a distinctive peak at ~1180 cm^−1^. Fraction 6 was notably enriched in signals within the ~704–732 cm^−1^ range, with a particularly prominent feature at ~718–722 cm^−1^.

To enable accurate interpretation of the bacterial fraction spectra, the Raman spectrum of the original culture medium without bacteria was recorded. The medium consisted of α-MEM (Biolot, Russia) supplemented with 0.2 mM myo-inositol, 0.02 mM folic acid, and 2 mM L-glutamine.

**Figure A10:**
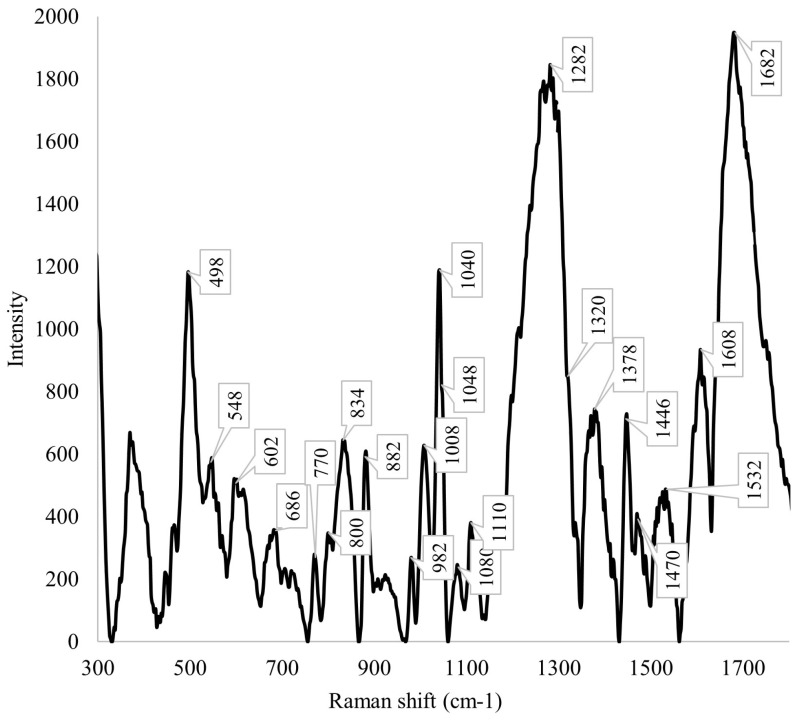
Raman spectrum of the culture medium for *Klebsiella pneumoniae* in the range of 300–3400 cm^−1^ [54].

The following peaks were detected in the medium spectrum (Figure A10) [54]: ~1682, ~1608, ~1532, ~1470, ~1446, ~1378, ~1320, ~1282, ~1110, ~1080, ~1048, ~1040, ~1008, ~982, ~882, ~834, ~800, ~770, ~686, ~602, ~548, and ~498 cm^−1^.

Assignment of these spectral signals, including their correspondence to individual components of the culture medium, was addressed in our previous study on the combined approach of chromatographic fractionation and Raman spectroscopy for metabolite profiling of *Enterobacter* spp. supernatant [54]. The cited publication provides a detailed interpretation of the culture medium’s Raman peaks, enabling the present work to focus specifically on identifying signals unambiguously associated with bacterial metabolites. In this work, powdered and crystalline myo-inositol were additionally analyzed by Raman spectroscopy, revealing characteristic bands at approximately ~1357 cm^−1^, ~1325 cm^−1^, ~1279 cm^−1^, ~1119 cm^−1^, ~1063 cm^−1^, ~1008 cm^−1^, ~932 cm^−1^, ~897 cm^−1^, ~734 cm^−1^, ~612 cm^−1^, ~503 cm^−1^, ~462 cm^−1^, ~428 cm^−1^, ~388 cm^−1^, and ~306 cm^−1^.

Thus, chromatographic fractionation enabled the identification of Fraction 3 as the most enriched and spectrally distinct, and further revealed several fraction-specific markers that were absent in both the US and the culture medium.

## 3. Discussion

Our data demonstrate that the combination of chromatographic fractionation and Raman spectroscopy enables effective deconvolution of the complex secretome of K. pneumoniae and facilitates the identification of fractions with unique metabolite profiles. Of particular interest is Fraction 3, whose spectral signature markedly differs from all other fractions and indicates enrichment with functionally significant biomolecules.

A key observation is the absence of the peak at ~1614–1618 cm^−1^ in Fraction 3, while it is present in all other fractions and the US. In the literature, this spectral region is traditionally attributed to aromatic amino acids—primarily tryptophan—and is associated with indole ring vibrations or asymmetric NH_3_^+^ bending modes [24]. Li et al. showed that upon UV irradiation of *Escherichia coli*, the intensity of the tryptophan peak at 1615 cm^−1^ systematically decreases, which the authors link to protein denaturation and conformational disruption of tryptophan residues [24]. Thus, the dynamics of the 1615 cm^−1^ peak serve not only as a marker of supposed tryptophan presence but also as a sensitive indicator of bacterial viability and cellular integrity. Reduced intensity correlates with increased cell death, making this peak a valuable diagnostic tool for rapid assessment of antimicrobial or sterilization efficacy. The absence of this signal in Fraction 3 may indicate either low abundance or structural rearrangement of tryptophan-containing proteins, rendering their functional groups inaccessible to Raman identification. In contrast, the pronounced peak in Fraction 6 (the most intense among all) and its moderate expression in Fractions 1, 2, 4, 7, and the supernatant suggest the presence of specific tryptophan-dependent components potentially linked to metabolic activity or stress response.

We propose that the absence of tryptophan markers in Fraction 3 reflects selective partitioning of hydrophobic, tryptophan-rich proteins into high-molecular-weight fractions, while Fraction 3 enriches soluble, tryptophan-poor proteins consistent with its distinct spectral profile. This pattern may also indicate metabolic adaptation, where K. pneumoniae modulates tryptophan-dependent pathways as an immune evasion strategy, suggesting Fraction 3 contains functionally active, tryptophan-independent effectors involved in stress response or early infection processes [4,5,6,7,8,9,10,11,12,24].

In the spectrum of K. pneumoniae Fraction 3, a prominent peak is observed at ~1672 cm^−1^, representing the most intense feature within the ~1670–1676 cm^−1^ range. All other fractions exhibit lower intensities in this region, with the weakest signal in Fraction 4. According to Pezzotti, the peak at ~1672 cm^−1^ can be confidently assigned to sphingomyelin (SPH), a sphingolipid whose amide-like band replaces the ester band (~1740 cm^−1^) typical of glycerophospholipids [21]. This band arises from C=O stretching coupled with N–H bending vibrations in the ceramide backbone [21]. Supporting this, Saraeva et al. identified the ~1676 cm^−1^ peak as a diagnostic marker of the Amide I band in proteins [22]. Further refinement by Varotsis et al., based on Fourier-transform infrared (FTIR) spectroscopy, attributes signals in the ~1670–1682 cm^−1^ range to β-turns in protein secondary structure [23]. Therefore, the strong ~1672 cm^−1^ peak in Fraction 3 suggests significant enrichment with either sphingolipids, β-turn-rich proteins, or a combination thereof.

Peaks in the ~1510–1526 cm^−1^ range are commonly associated with the Amide II band, arising from N–H bending and C–N stretching in the peptide backbone [26]. This band is a sensitive indicator of protein secondary structure and relative abundance [26]. The high intensity of this peak in Fraction 3 likely reflects elevated protein content or structural reorganization compared to other fractions. Kochan et al. reported that Amide II intensity shifts correlate with metabolic adaptation in bacteria, consistent with the peptide- and protein-metabolite enrichment observed in Fraction 3 [26]. Additionally, contributions from tyrosine (~1515 cm^−1^) [21], cytochrome c porphyrin ring vibrations (~1518 cm^−1^) [28], and O-antigen polysaccharides (~1523–1688 cm^−1^, via C=O and C–O–H deformations) [29] cannot be excluded. Thus, the enhanced signal in this region likely reflects a composite contribution from proteins, heme-containing molecules, and O-polysaccharides.

The ~1460–1476 cm^−1^ region is typically assigned to CH_2_ deformation (δ(CH_2_)) and symmetric C–H stretching (νs(CH_2_)) in saturated lipids [21,30]. Gieroba et al. confirmed this assignment as νs(CH_2_) for saturated lipids [30], while Pezzotti linked the ~1465 cm^−1^ signal in glycerol to CH_2_ bending, underscoring its relevance to glycerol-containing lipids [21]. Borisova et al. identified ~1468 cm^−1^ as a key SERS marker for differentiating bacterial strains, interpreted as δ(CH_2_) of saturated fatty acids [31]. Notably, this region may also include signals from osmoprotectants (e.g., ectoine at ~1469 cm^−1^, glycine betaine at ~1460–1462 cm^−1^) [32] and cell wall components, likely peptidoglycan and teichoic acids [21]. The uniquely high intensity at ~1476 cm^−1^ in Fraction 2 suggests selective enrichment with lipid acyl chains or ordered hydrophobic protein domains.

A distinctive peak at ~1382–1410 cm^−1^ is exclusive to Fraction 3. Dastgir et al. assigned the ~1402 cm^−1^ band to carboxylate (COO^−^) stretching in amino acids based on SERS qualitative analysis [33], corroborated by Anwer et al., who linked ~1404 cm^−1^ to α-amino acid N(CO_2_^−^) stretching in proteins [34]. This vibration is sensitive to ionization state, hydrogen bonding, and metal coordination [21]. Saraeva et al. observed broadening and red-shifting of the ~1396 cm^−1^ COO^−^ band after bacterial electroporation, indicating peptidoglycan disruption [22]. Guicheteau et al., using SERS, further associated this region with aspartic/glutamic acid side chains and pyrimidine ring C–N stretching [35], while Tahir et al. linked ~1396 cm^−1^ to N-acetylglucosamine in peptidoglycan [36]. Thus, this peak likely reflects a composite of free amino acids, peptide fragments, nucleotides, and cell wall components.

In all *K. pneumoniae* fractions except Fractions 3 and 4, a pronounced peak is observed in the range ~1366–1370 cm^−1^. According to Jehlicka et al., the signal at ~1366 cm^−1^ can be attributed to NH^+^ deformation vibrations in the zwitterionic structure of ectoine, as well as δ(CH_2_) bending modes in glucosylglycerol and other glycerol-containing osmoprotectants [32]. The peak at ~1371 cm^−1^, reported for di-myo-inositol phosphate, has been assigned to symmetric CH_2_ deformation vibrations in aliphatic chains [32]. Further insight into this spectral region is provided by Guicheteau et al., who characterized Raman signatures of amino acids and nucleotide bases [35]. Their study identified the ~1365 cm^−1^ peak as characteristic of aromatic ring stretching modes of phenylalanine and tyrosine, while signals at ~1371–1373 cm^−1^ were linked to CH_3_/CH_2_ deformation vibrations in aliphatic amino acids such as alanine, leucine, and glutamic acid. The absence of this signal in Fractions 3 and 4 is particularly noteworthy and may indicate a unique biochemical composition depleted in ectoine, glucosylglycerin, or other osmoprotective compounds.

Comprehensive analysis reveals distinct peaks in the ~1342–1352 cm^−1^ region in the spectra of K. pneumoniae Fractions 3 and 4. Fraction 3 exhibits a strong signal at ~1342 cm^−1^, whereas in Fraction 4 the peak shifts to ~1352 cm^−1^. Guicheteau et al. assigned the ~1342 cm^−1^ band to CH deformation and/or symmetric COO^−^ stretching in leucine, while the ~1352 cm^−1^ signal corresponds to analogous vibrational modes in methionine [35]. These findings align with interpretations by McEwen et al. and Pezzotti, who consider this spectral region a sensitive marker for δ(CH_2_) deformations in proteins and polysaccharides [21,37]. Osorio-Román et al. further associated SERS signals in the 1252–1352 cm^−1^ range with C–H deformation vibrations in O-polysaccharides of *E. coli* and *Salmonella* spp. [29]. The slight shift from ~1342 cm^−1^ to ~1352 cm^−1^ likely reflects changes in the local microenvironment of the vibrating groups, potentially indicating a transition from a hydrophilic milieu (soluble proteins/nucleic acids) to a more hydrophobic one (membrane domains or aggregated polysaccharides), or a shift in amino acid composition (leucine → methionine) contributing to the spectral signature.

A prominent peak at ~1286 cm^−1^ is observed in the spectrum of *K. pneumoniae* Fraction 3. While present in other fractions, its intensity is substantially lower elsewhere. Literature data assign this region to the Amide III band of the protein backbone (C–N stretching coupled with N–H deformation) or ring-breathing vibrations of benzene (proteins/peptides) [21,35,37]. Signal enhancement in Fraction 3 may indicate elevated levels of proteins with specific secondary structures, such as β-sheet conformations, which shift the Amide III band into this region, or accumulation of lipids with highly ordered hydrocarbon chains [37]. Given that separation was performed under non-denaturing conditions via Size Exclusion Chromatography (SEC), this fraction is likely enriched in high-molecular-weight protein-lipid complexes.

Fractions 4 and 5 exhibit a distinct and unique peak at ~1180 cm^−1^, absent in all other fractions and the US. This signal may arise from C–N–C antisymmetric stretching vibrations of secondary amines characteristic of proline [35], or from C_6_H_5_–C/phenyl C-stretching modes in phenylalanine [21,38]. The literature also reports that the ~1172–1199 cm^−1^ range corresponds to CH_2_ twisting and rocking deformations in amino acid side chains [35,39]. The exclusive presence of this peak in Fractions 4 and 5 suggests specific accumulation of protein components enriched in proline or aromatic amino acids, potentially linked to activated secretory pathways or formation of extracellular protein complexes under the applied culture conditions. It is necessary to consider that the signal from proline and aromatic amino acids is difficult to separate.

Broadened bands in the ~1000–1200 cm^−1^ region were detected across the US and its chromatographic fractions. The most intense signal in Fraction 3 occurred at ~1132 cm^−1^. According to the literature, vibrations in this interval may originate from multiple biomolecule classes: (i) the ~1128 cm^−1^ band is characteristic of C–C skeletal stretching in lipid backbones [21,22]; SERS studies further interpret the ~1132 cm^−1^ peak as arising from unsaturated fatty acids [34]; (ii) symmetric PO_2_^−^ stretching vibrations of phospholipids and nucleic acids appear in the ~1070–1090 cm^−1^ range and often overlap with DNA/RNA signals [21,22]; (iii) Guicheteau et al. [35] report that the ~1120–1152 cm^−1^ interval includes C–C skeletal stretching modes of amino acids and nucleotide bases. Additional contributions may derive from deoxynucleoside triphosphates (dNTPs): Pezzotti [21] notes C–H deformations of dATP at ~1130 cm^−1^ and a dCTP doublet at ~1102/~1163 cm^−1^. Due to significant spectral overlap among lipids, proteins, nucleic acids, dNTPs, and catecholamines, bands in the ~1128–1148 cm^−1^ range are generally considered unsuitable for unambiguous biomolecule classification by conventional Raman spectroscopy [21,22].

Fractions 1 and 2 display two well-resolved peaks at ~1090 cm^−1^ and ~1084 cm^−1^, absent in other fractions and the supernatant, making them useful vibrational markers for sample differentiation. The ~1080–1100 cm^−1^ range in bacterial Raman spectra is traditionally associated with PO_2_^−^ stretching vibrations of nucleic acid phosphodiester bonds and C–O vibrations of cell wall carbohydrates [26,35]. Specifically, the ~1098 cm^−1^ band is interpreted as a DNA component [40], while nucleotide triphosphate spectra show O–P–O phosphate backbone vibrations in this region [34]. Saraeva et al. [22] confirmed that the ~1085 cm^−1^ PO_2_^−^ band shifts to ~1090 cm^−1^ upon DNA/RNA structural alterations, accompanied by signal intensity reduction. Pezzotti [21] notes that symmetric PO_2_^−^ stretching in phospholipids also occurs in the ~1070–1090 cm^−1^ range and may overlap with nucleic acid signals. Additional contributions may include C–C skeletal vibrations of lipid backbones (~1060–1095 cm^−1^) [35] and sphingo-myelin acyl chain signals (~1090 cm^−1^). Protein contributions are also plausible: studies on the DNA-binding HU protein from Bacillus stearothermophilus assigned the ~1082 cm^−1^ band to combined C–C and C–N skeletal vibrations of the arginine guanidinium group [41]. The presence of two distinct peaks (~1084 and ~1090 cm^−1^), rather than a single shifted maximum, suggests coexisting spectrally resolvable molecular populations: one corresponding to nucleic acid/phospholipid phosphodiesters (~1090 cm^−1^), the other to arginine-rich protein domains or specific biopolymer conformations.

Fraction 3 exhibits a pronounced peak at ~1044 cm^−1^, with intensity substantially exceeding that in other fractions. Although the literature mentions vibrational modes in this vicinity, particularly C–O–C and C–C stretching in carbohydrates (~1043 cm^−1^) [42,43,44] or cell wall lipid contributions [45], no available data unambiguously assign peaks in the narrow ~1042–1050 cm^−1^ range to Klebsiella-specific metabolites or structural components. Therefore, definitive attribution of this signal requires additional studies employing chromatography-mass spectrometry or isotopic labeling to verify the molecular nature of the observed vibration.

In the spectrum of K. pneumoniae Fraction 7, distinct and unique peaks are observed in the range of ~1020–1026 cm^−1^, which are absent in all other fractions and the whole cell-free supernatant. These signals serve as key markers distinguishing Fraction 7 from all others. According to Pezzotti et al., the border signal seen at ~1026 cm^−1^ arises from NH2 rocking. Based on SERS measurements, vibrations in this spectral region may arise from C–C skeletal stretching modes of aromatic amino acids, particularly phenylalanine (peak at ~1029 cm^−1^) [35], as well as O–P–O stretching vibrations of phosphate groups in nucleic acids (e.g., ~1028 cm^−1^ for adenosine) [35]. However, such vibrations typically appear within a broader spectral context and are accompanied by characteristic secondary bands associated with protein or nucleic acid structures. At the time of writing, no data in the available literature allow unambiguous assignment of peaks in the ~1020–1026 cm^−1^ range specifically to bacterial metabolites or cellular components of K. pneumoniae. Thus, the observed signals likely represent either previously uncharacterized vibrational modes of Fraction 7, –S–S– specific metabolites, or conformational alterations of known biomolecules under the experimental conditions employed.

Additionally, peaks at ~992 cm^−1^ and ~1000 cm^−1^ are detected exclusively in Fraction 3 and are absent in all other samples. The signal in the ~1000–1002 cm^−1^ region is traditionally interpreted in biological systems as the ring-breathing vibration of the benzene ring in phenylalanine [22,35], suggesting elevated levels of aromatic amino acids or phenylalanine-enriched protein domains. In contrast, the peak at ~992 cm^−1^ may correspond to C–C or C–O–C skeletal vibrations in carbohydrate fragments or reflect conformational features of the peptide backbone [21]. The absence of these signals in other fractions indicates qualitative differences in molecular composition and may point to the specific localization of protein-carbohydrate complexes or metabolites unique to Fraction 3.

A pronounced peak at ~876 cm^−1^ is observed in Fractions 1 and 3 of K. pneumoniae. While present in other fractions, its intensity is substantially lower elsewhere. This band is close in position to the ~873–874 cm^−1^ signal reported in bone Raman spectroscopy and attributed to proline and hydroxyproline vibrations in collagen matrices [46]. In bacterial spectra, analogous bands may reflect contributions from cell wall proteins or extracellular polymers rich in amino acids. In studies of Pseudomonas putida KT2440, a peak at ~874 cm^−1^ was also detected in early-cultivation-phase cells (4–6 h) and preliminarily assigned to C–C stretching (ν(C–C)) in extracellular polymeric substances [37]. Furthermore, in membrane Raman spectroscopy, the ~876 cm^−1^ band is attributed to antisymmetric C–N stretching in the N^+^(CH_3_)_3_ groups of choline headgroups in phospholipids, specifically phosphatidylcholine and sphingomyelin [21]. In this context, variations in the intensity of this signal may reflect changes in the composition or structural organization of bacterial membrane lipids.

Fraction 3 exhibits a strong peak at ~814 cm^−1^, which is also present, though significantly weaker, in other fractions and the US. Literature data assign vibrations in the ~810–830 cm^−1^ range to aromatic ring deformations of phenylalanine and tyrosine, as well as C–S stretching in methionine [22,35]. Specifically, the ~823 cm^−1^ peak is associated with C–S stretching in methionine and ring deformation of phenylalanine/tyrosine [35], while the ~829 cm^−1^ signal is characteristic of para-substituted benzene rings in tyrosine [35]. Notably, in growth dynamics studies of Cupriavidus metallidurans, a peak at ~808 cm^−1^ was detected only in differential spectra between growth stages, indicating its association with metabolites whose concentrations vary during the cell cycle [21]. The presence of these signals in bacterial supernatants suggests secretion of proteins enriched in aromatic and sulfur-containing amino acids.

Moreover, Fraction 3 displays a unique peak at ~772 cm^−1^, absent in all other fractions. According to Carey et al., peaks in the ~770–773 cm^−1^ range belong to a group of “new bands” reproducibly observed in bacterial Raman difference spectra under antibiotic exposure or oxidative stress [47]. The authors interpret these signals as reflecting shifts in intracellular metabolite populations, particularly implicating citrate, a tri-carboxylic acid (TCA) cycle intermediate. This assignment is supported by the absence of the ~770 cm^−1^ band in spectra of an E. coli gltA mutant incapable of citrate synthesis [47]. Additionally, the intensity of the ~770 cm^−1^ peak decreases over time, suggesting dynamic metabolic remodeling in response to external stressors [47]. Thus, the presence of the ~772 cm^−1^ peak in Fraction 3 likely indicates specific accumulation of stress-responsive metabolites.

In Fraction 6, a prominent peak appears in the ~704–732 cm^−1^ range, with intensity markedly exceeding that in other fractions or being entirely absent elsewhere. This spectral region is of particular interest, as it encompasses characteristic vibrational modes of key biomolecules. Guicheteau et al. [35] associate peaks at ~729–732 cm^−1^ with ring-breathing vibrations of pyrimidine bases in nucleic acids (ATP, TTP, UTP), while signals near ~719 cm^−1^ correspond to S–CH_3_ stretching in methionine. The broad band observed in Fractions 1, 2, and 5–7 (~704–732 cm^−1^) likely results from overlapping contributions of aromatic amino acids (phenylalanine, tyrosine, tryptophan) [22,35], pyrimidine bases, and sulfur-containing compounds. The complete absence of this signal in Fraction 3 underscores its distinct molecular composition. Conversely, the maximal intensity in Fraction 6 (~718–722 cm^−1^) suggests enrichment with nucleotide components and/or sulfur-containing protein metabolites.

Notably, Fraction 3 shows a pronounced peak at ~664 cm^−1^, also present, but significantly weaker, in other fractions and the US. Literature associates the ~660–670 cm^−1^ region with guanine ring vibrations—a purine base in DNA and RNA [35,47]. Oliveira et al. (2021) reported enhanced ~667 cm^−1^ intensity in clinical isolates of K. pneumoniae and P. aeruginosa, correlating with high genomic GC (guanine-cytosine) content (57.1% and 66.2%, respectively) [20]. Guicheteau et al. further note that peaks at ~664–666 cm^−1^ may also arise from C–S stretching in methionine [35]. Carey et al. confirmed that the ~667 cm^−1^ band in antibiotic-treated E. coli corresponds to guanine ring vibrations in nucleic acids [47]. The enhanced ~664 cm^−1^ signal in Fraction 3 thus indicates enrichment with nucleic acids, nucleotide-containing components, or methionine-rich protein structures.

In the spectrum of K. pneumoniae Fraction 3, a pronounced peak is observed at ~596 cm^−1^, with intensity substantially exceeding that in all other fractions and the US. According to the literature, the ~590–605 cm^−1^ region in bacterial Raman spectra is attributed to deformation vibrations of purine and pyrimidine bases in DNA/RNA, particularly cytosine and thymine [21,35]. Pezzotti notes that in the low-frequency range ~600–700 cm^−1^, which is free from lipid contributions, characteristic signals of deoxynucleoside triphosphates (dNTPs) are observed: the peak at ~600 cm^−1^ corresponds to C=O deformation modes in dCTP, while signals at ~648–650 cm^−1^ are assigned to ring deformations of adenine and guanine in dATP and dGTP [21,25]. Additionally, Guicheteau et al. report that the band at ~596 cm^−1^ may also arise from aromatic ring deformations of phenylalanine or tyrosine, as well as from coupled modes of the peptide backbone [35]. The pronounced enhancement of the ~596 cm^−1^ signal specifically in Fraction 3 suggests enrichment with nucleotide-containing components, extracellular DNA/RNA fragments, or peptides rich in aromatic amino acids [25].

Fraction 3 also exhibits a distinct and unique peak at ~532 cm^−1^, which is absent or significantly weaker in all other samples. According to Brandt et al., the ~528–538 cm^−1^ spectral range in biological Raman spectra can be assigned to S–S stretching vibrations of cysteine disulfide bonds [50]. In model bacterial cultures (Bacillus globigii, Pantoea agglomerans, Yersinia rhodei), signals in the ~529–549 cm^−1^ region have been interpreted as contributions from sulfur-containing amino acids or amide-containing components [35]. The enhanced intensity at ~532 cm^−1^ in Fraction 3 thus likely indicates enrichment with peptides or proteins highly enriched in cysteine, asparagine, or glutamine.

In contrast, Fraction 2 displays a strong peak at ~486 cm^−1^, with intensity markedly higher than in all other fractions and the cell-free supernatant. Ehsan et al. assigned the band at ~484 cm^−1^ in SERS spectra of human serum to uric acid—a metabolite whose levels increase during cellular damage and purine metabolism disruption in infectious conditions [49,51]. Furthermore, a peak near ~490 cm^−1^ may correspond to S–S stretching vibrations of cysteine in proteins [49,51]. In the context of bacterial cultures, such a signal may reflect accumulation of nucleic acid degradation products or specific low-molecular-weight metabolites released during cell lysis or active secretion. The selective enhancement of the ~486 cm^−1^ signal in Fraction 2 suggests enrichment with low-molecular-weight compounds (<30 kDa), such as oligonucleotides, peptides, or organic acids.

Finally, the peak in the ~384–386 cm^−1^ region falls within the low-frequency Raman range, typically associated with skeletal deformations, phosphate group vibrations in nucleic acids, or vibrations linked to sulfur-containing moieties (C–S, S–S) [52,53]. The slight shift in this peak—~384 cm^−1^ in Fractions 2, 3, 5–7 versus ~386 cm^−1^ in Fractions 1 and 4—may reflect differences in the local chemical environment or conformational states of the signal-bearing molecules. The maximal intensity observed in Fraction 3 further supports its enrichment with a specific metabolite or protein component bearing these structural features.

## 4. Materials and Methods

### 4.1. Bacterial Strain and Culture Conditions

We used *Klebsiella pneumoniae* strain ATCC 13882. Bacteria were cultured on agar plates under appropriate biosafety containment, in accordance with institutional protocols for handling pathogenic microorganisms.

### 4.2. Preparation of Conditioned Media from ESKAPE Bacteria

To prepare conditioned media, bacterial cultures were inoculated into α-MEM medium (Biolot, Saint-Petersburg, Russia) supplemented with 0.2 mM myo-inositol (Sigma, St. Louis, MO, USA (Product of Chine)), 0.02 mM folic acid (Sigma, USA (Product of Chine)), and 2 mM L-glutamine (Sigma, USA (Product of Chine)) to a final concentration of 1 × 10^8^ CFU/mL. Cultivation was carried out in sterile glass vials containing 5 mL of medium for 24 h at 37 °C under 5% CO_2_ atmosphere. Following incubation, the supernatant was filtered through 0.45-μm pore-size syringe filters (Sarstedt, Nümbrecht, Germany) to remove bacterial cells.

### 4.3. Size Exclusion Chromatography (SEC)

Cell-free bacterial supernatants were concentrated using a CentriVap Vacuum Concentrator (Labconco Corp., Kansas City, MI, USA) to a volume yielding a total protein concentration exceeding 5 mg/mL. Fractionation was performed using an Agilent 1260 Infinity II high-performance liquid chromatography system equipped with OpenLAB CDS ChemStation software (Workstation version A.02.02) (Agilent Technologies, Inc., Santa Clara, CA, USA) (Figure A1). Separation was carried out under non-denaturing conditions on an Agilent Bio SEC-3 size-exclusion column (3 μm, 300 Å, 4.6 × 300 mm; Agilent Technologies, Inc., Santa Clara, CA, USA), with bi-distilled water as the mobile phase. The analysis was conducted in isocratic mode at 4 °C and a flow rate of 0.35 mL/min. A diode array detector monitored absorbance at 210, 230, 254, and 280 nm over a 30-min run. Due to the continuous elution profile, fractions were collected in 3-min intervals starting from minute 3. No protein was detected after minute 24, and this late-eluting material was excluded from further analysis. The collected fractions were reconcentrated to 2.5 mL using the CentriVap Vacuum Concentrator to standardize sample volume, as protein concentrations were below the detection limit. Fractions were then filtered through 0.45-μm syringe filters (Corning Inc., Corning, New York, NY, USA), frozen at −80 °C, and stored for up to two weeks prior to analysis.

### 4.4. Raman Spectroscopy

Raman spectra were acquired using an OPTEC 785LRam spectrometer (OPTEC, Saint-Petersburg, Russia) equipped with a 785-nm laser (power adjustable between 10 and 300 mW). The spectral range spanned 300–3400 cm^−1^ with a resolution of 8 cm^−1^. Samples were analyzed in the liquid phase in 2-mL glass vials. At least three independent spectra were recorded per sample, each averaged over 100 scans. Prior to measurement, samples were equilibrated to room temperature and thoroughly mixed. Baseline correction was performed using iterative polynomial approximation. Spectral data were processed using BWSpec4 and OPTEC-Raman v1.2 software.

## 5. Conclusions

Comparison of the spectra of the US and chromatographic fractions with the background spectrum of the pure culture medium enabled the identification of signals associated with bacterial metabolites. Since the intensities of medium-specific peaks were consistently lower in bacterial samples than in the control, the observed spectral contributions are predominantly attributed to bacterial metabolic products. Nevertheless, definitive verification of the molecular origin of these bands requires further investigation.

Two distinct groups of Raman signals were identified in the whole cell-free supernatant of *K. pneumoniae* and its fractions: medium-derived peaks (~384 cm^−1^, ~602 cm^−1^, ~1048 cm^−1^, ~1282 cm^−1^, ~1466–1474 cm^−1^, ~1682 cm^−1^), which likely reflect residual components of the culture medium whose signals are attenuated or masked during chromatographic separation, and putative markers of bacterial metabolism (absent in the pure medium spectrum): ~386 cm^−1^, ~484 cm^−1^, ~490 cm^−1^, ~528–534 cm^−1^, ~596 cm^−1^, ~664–672 cm^−1^, ~704–732 cm^−1^, ~772 cm^−1^, ~808–826 cm^−1^, ~872–876 cm^−1^, ~916–930 cm^−1^, ~992 cm^−1^, ~1000 cm^−1^, ~1020–1026 cm^−1^, ~1042–1050 cm^−1^, ~1082–1098 cm^−1^, ~1124–1148 cm^−1^, ~1180 cm^−1^, ~1284–1292 cm^−1^, ~1342 cm^−1^, ~1352 cm^−1^, ~1366–1370 cm^−1^, ~1382–1410 cm^−1^, ~1460 cm^−1^, ~1476 cm^−1^, ~1480 cm^−1^, ~1510–1526 cm^−1^, ~1614–1618 cm^−1^, ~1670–1676 cm^−1^.

The combined approach of chromatographic fractionation and Raman spectroscopy successfully deconvoluted the complex secretome of the Gram-negative pathogen *K. pneumoniae*, overcoming the challenge of spectral congestion. A key finding was the identification of Fraction 3 as uniquely distinct in its metabolite profile: it lacks markers of tryptophan (~1614–1618 cm^−1^) and sterol-like lipids (~548 cm^−1^), sharply differentiating it from all other fractions and the US. This fraction exhibits maximal signal intensity indicative of enrichment with functionally significant biomolecules; specifically, peptides containing tyrosine and methionine (~1510–1526 cm^−1^, ~850/830 cm^−1^, ~814 cm^−1^), nucleic acid fragments (~1080 cm^−1^, ~1110 cm^−1^, ~664 cm^−1^), polysaccharides (~1044 cm^−1^), and stress-response metabolites such as citrate (~772 cm^−1^). The presence of a signal at ~1014 cm^−1^ may indicate glutathione-like compounds or peptides with a similar C–C scaffold structure, underscoring the potential role of this fraction in protection against oxidative stress.

Comparative analysis of spectral profiles across fractions revealed their specific biochemical specialization: Fraction 2 is enriched in uric acid (~486 cm^−1^) and saturated lipids (~1476 cm^−1^); Fraction 6 contains nucleotide components and sulfur-containing metabolites (~704–732 cm^−1^); Fractions 4 and 5 are characterized by proline- and phenylalanine-containing peptides (~1180 cm^−1^); Fraction 5 is enriched in pyrimidine bases (~1478 cm^−1^, ~1488 cm^−1^); Fraction 7 harbors unique metabolites in the ~1020–1026 cm^−1^ range, whose molecular nature warrants further investigation.

Furthermore, the shift of the tryptophan peak from ~880 cm^−1^ (indicative of a hydrophilic, solvent-exposed environment) to ~876 cm^−1^ (reflecting a hydrophobic conformation within lipid domains or protein aggregates) across fractions highlights differences in the microenvironment and topology of secreted components.

These findings confirm that size-exclusion chromatography (SEC) effectively reduces spectral complexity, enabling Raman spectroscopy to resolve unique “metabolic fingerprints” of individual fractions. Detailed characterization of the *K. pneumoniae* metabolite profile is critical for deciphering mechanisms of pathogenicity and antibiotic resistance. Secreted metabolites mediate key survival processes, including intercellular communication, biofilm formation, host immune modulation, and antibiotic resistance mechanisms. Identification of specific spectral signatures, such as stress-response markers (e.g., citrate) or extracellular matrix components, allows functional assessment of bacterial population states and detection of potential virulence factors in their native conformation. Although final molecular identification requires complementary approaches such as mass spectrometry and enzymatic digestion, the proposed hybrid methodology opens new avenues for functional screening of the bacterial secretome.

## Figures and Tables

**Table 1:** Assignment of Raman peaks detected in this study to molecular structures.

Raman Shift (cm^−1^)	Molecular Assignment	Detected in	References
~1682–1676	Amide I (C=O stretching in peptide bonds of proteins and peptides)	Fractions 1, 2, 4, 5	[20]
~1672–1670	Amide-like band of sphingomyelin (C=O stretching + N–H deformation); β-turns in proteins	US, fractions 3, 6, 7	[21,22,23]
~1614–1618	Ring-breathing vibrations of tryptophan indole ring; asymmetric bending of NH3+ group	US, fractions 1, 2, 4–7	[24,25]
~1510–1526	Amide II band (N–H bending + C–H stretching in peptide backbone); tyrosine; cytochrome c	US, fractions 1–7	[21,24,26,27,28,29]
~1480–1476	CH_2_ deformation (δ(CH_2_)) in saturated lipids; pyrimidine bases	Fractions 2, 5–7	[24,30,31,32,33]
~1460–1474	Symmetric C–H stretching (ν(CH)) in saturated lipids; peptidoglycan; osmoprotectants	US, fractions 1, 3, 4	[24,30,31,32]
~1382–1410	Carboxylate (COO^−^) stretching; C–H stretching in amino acids; peptidoglycan; assignoble to carboxylate/amino acid modes	Fraction 3	[21,24,30,31,32,33,34,35,36]
~1366–1370	NH^+^ deformation in ectoine; δ(CH_2_) in glucosylglycerol; aromatic amino acids	US, fractions 1, 2, 5–7	[24,30,32,35]
~1352	C–H deformation and symmetric COO^−^ stretching in methionine; O-polysaccharides	Fraction 4	[21,29,37]
~1342	C–H deformation in nucleic acid and symmetric COO^−^ stretching in leucine	Fraction 3	[21,29,37]
~1282–1286	Amide III band (C–N stretching + N–H deformation); ring-breathing vibrations of benzene (proteins/peptides)	US, fractions 1–7	[21,35,37]
~1180	C–H–C asymmetric stretching in secondary amines (proline); C_6_H_5_–C stretching (phenylalanine)	Fractions 4, 5	[21,35,38,39]
~1126–1128	C–C skeletal stretching in lipid backbone; unsaturated fatty acids; glycosidic bond (C–O–C)	Fraction 3	[21,22,35]
~1090–1098 and ~1082–1084	Symmetric PO_2_^−^ stretching in nucleic acids; arginine-containing proteins	US, fractions 1–7	[21,22,26,35,40,41]
~1080–1082	Symmetric phosphate (PO_2_^−^) stretching in nucleic acids and phospholipids	Fractions 6, 7	[21,22,26,35,40,41]
~1042–1050	C–O–C and C–C stretching vibrations in carbohydrates; cell wall lipids	US, fractions 1–7	[42,43,44,45]
~1020–1026	NH_2_ rocking	Fraction 7	[21]
~992 and ~1000	Benzene ring breathing mode of phenylalanine; C–C or C–O–C skeletal vibrations in carbohydrates	Fraction 3	[21,22,35]
~930	Unidentified band	US	–
~916–926	Unidentified band	US, fractions 1–7	–
~876	Antisymmetric C–H stretching in choline headgroups of phospholipids	Fractions 1, 3, 4	[21,37,46]
~872–874	C–C stretching (ν(C–C)) in extracellular polymeric substances	US, fractions 2, 5–7	[21,37,46]
~814–826	Aromatic ring deformations of phenylalanine and tyrosine; C–S stretching in methionine	US, fractions 1–7	[21,22,35]
~772	“New band”—tricarboxylic acid cycle metabolites (citrate)	Fraction 3	[47]
~728	Ring deformation of adenine (purine base in DNA/RNA)	Fraction 7	[48]
~704–732	Pyrimidine ring breathing in nucleic acids; –CH_3_ stretching in methionine; aromatic amino acids	US, fractions 1–7	[22,45]
~664–672	Purine ring vibrations of guanine; C–S stretching in methionine	US, fractions 1–7	[20,35,47]
~602	Unidentified band	US, fractions 4–6	–
~596	Deformation of purine/pyrimidine bases (cytosine, thymine); aromatic amino acids	Fractions 1–3, 7	[21,25,35]
~528–532	Skeletal O=C– deformation in asparagine and L-glutamine side chains; disulfide (–S–S–) stretching in cysteine	US, fractions 1, 3–6	[49]
~486–490	Uric acid vibrations; –S– stretching in cysteine	US, fractions 1–5	[49,50,51]
~484	Unidentified band	Fractions 6, 7	–
~384–386	Skeletal deformations; phosphate group vibrations in nucleic acids; sulfur-containing compounds (C–S, S–S)	US, fractions 1–7	[52,53]

## Data Availability

The original contributions presented in this study are included in the article. Further inquiries can be directed to the corresponding author.
